# Maternal Mortality and the Public Health Service in Brazil

**DOI:** 10.1055/s-0041-1736537

**Published:** 2021-10-20

**Authors:** Marcos Felipe Silva de Sá

**Affiliations:** 1 Departamento de Ginecologia e Obstetrícia, Faculdade de Medicina de Ribeirão Preto, Universidade de São Paulo, Ribeirão Preto, SP, Brazil

Maternal mortality (MM) has been a serious problem for public health worldwide and the subject of many debates internationally. In addition to health issues, this indicator also reflects the social conditions of victimized women. Maternal mortality represents a family and social tragedy, and many of these deaths result from avoidable causes. About 70% are caused by complications such as bleeding, infections, unsafe abortions, eclampsia and labor dystocia, which makes the statistics a reflex of the quality of women's health care. Nowadays, 830 women die in Brazil every day from preventable causes related to pregnancy and childbirth. According to the Brazilian Obstetric Observatory, 38 maternal deaths from Covid-19 were recorded each week in Brazil in 2021. 1

In 2000, at the Millennium Summit sponsored by the United Nations, a 75% reduction in maternal mortality rates was established as a goal in an agreement signed by the 191 member nations, adopting indicators of year 1990 as a starting point. 2 According to available data, MM in Brazil was of 143/100 thousand live births (LB) in 1990, despite the well-known underreporting of deaths. The goal to be reached in 2015 by Brazil would be 35.8 MM/100 thousand LB.

Exactly in 1990, Law No. 8080 was enacted, which created a new National Health Service in Brazil (Sistema Único de Saúde - SUS - acronym in Portuguese). This September, it completes 31 years of existence, 3 and SUS represented an enormous advance that revolutionized the public health service in the country. The MM theme was one of the first to be placed on the agenda of public health policies to be implemented. 4 Maternal deaths became of mandatory notification and this decision greatly improved the reliability of indicators. Maternal Mortality Committees (MMC) were created to investigating the circumstances, identifying the causes and the technical and administrative responsibilities of each case, an important step to define measures to remedy the irregularities. Maternal Mortality Committees (MMC) were created with the objective of investigating the circumstances, identifying the causes and the technical and administrative responsibilities of each case and, from there, define measures to remedy the irregularities. Maternal Mortality Committees also aim to promote educational and awareness-raising activities for the community and professionals involved in women's health care. There was a considerable improvement in the information about each occurrence and the MMC have been performing an important role in the control and monitoring of MM rates. 5 6 Undoubtedly, there has been considerable progress, as in 2015, Brazil reached the rate of 62.0 MM/100 thousand LB. However, there was a stagnation in the reduction process as of 2012 and unfortunately in 2017, Brazil presented 64.0 MM/100 thousand LB, with the greatest increase in the north and northeast regions, the poorest in the country. These data raised an alert for health managers and professionals in Brazil, considering that in a such sense, these indicators reflect the precariousness of maternal and child health care in the period.

Between 1996 and 2018, 38,919 maternal deaths were registered, of which approximately 67% originated directly from obstetric complications during pregnancy, childbirth or puerperal period, due to interventions, omissions, incorrect treatment or a chain of events resulting from any of these causes. 7 In 2018, the rate was of 59.1 MM/100 thousand LB. Therefore Brazil is far from a comfortable situation compared to data from Japan (5 MM/100 thousand LB), Denmark (4 MM/100 thousand LB), United States (19 MM/100 thousand LB) and Spain (4 MM/100 thousand LB).

There is now, again, a worldwide alert, as the goals are not being met in many countries. This September, in celebration for the World Patient Safety Day, The World Health Organization (WHO) created the Safe Maternal and Neonatal Care campaign as the main theme. In Brazil, several civil entities joined immediately and the National Association for Safe and Respectful Childbirth was created ( http://aliancapartoseguro.org.br/ ). The Brazilian Federation of Gynecology and Obstetrics Associations – FEBRASGO, is a participant initiative. Actually, Febrasgo had already anticipated and created, a decade ago, the Specialized National Commission on Maternal Mortality with the objective of supporting the Ministry of Health of Brazil in the development of public policies to reduce maternal mortality. According to the Specialized National Commission on Maternal Mortality, in fact, the Ministry of Health's plan to reduce MM consists of numerous isolated initiatives; but the system fundamentally lacks a hierarchical organization of the patient flow to facilitate pregnant women's access to services. In general, deaths occur as a result of delays in providing appropriate care. Basically, the Specialized National Commission on Maternal Mortality refers to three important points: 1- misinformation often leads pregnant women to delay the decision to seek care; 2- access to the appropriate service location may be delayed due to transport logistics issues; 3- there may be delays in care at the reference institution. On many occasions, the patient still needs to be transferred from a place of care to another institution with a higher level of complexity, and this access is not always facilitated by an agile flow of referrals. 8

During the pandemic of Coronavirus-19, failures in the organization of the system became more evident, and Brazil emerged with one of the highest maternal mortality rates resulting from Covid-19. This fact is attributed to some factors such as barriers in accessing health services, regional differences in measures to contain the pandemic and the prevalence of concomitant risk factors that made the disease by Covid-19 more severe among pregnant women, increasing the annual number of maternal deaths in Brazil by 10%. 9 10 11 The path to a solution is already well known by health managers and professionals. The organization of health services focused at pregnant women should start with an access to qualified prenatal care, prevention of pregnancy-related diseases as well as to identify and planning of delivery in cases of greater risk and timely referral of more complex cases for assistance in places with better technical and infrastructure resources. However, it is not always possible to count on qualified professionals in the health services involved. It is essential to improve the quality and effectiveness of services with frequent training of those involved, preparing them for actions focused on women's care guided by the best scientific evidence.

A regionalized and hierarchical organization of the patient flow and the implementation of care networks with institutions appropriately structured to perform clinical and surgical procedures are necessary. The allocation of resources to medium-sized hospitals that can meet the demands should be prioritized, including the provision of ICU beds. Protocols must be prepared in accordance with the peculiarities of institutions, taking into account their level of complexity, the work in a multidisciplinary team with tasks well divided among all, in accordance with the established in a matrix of competences, with protocols to be followed for the whole team. 12 13

This September, the Brazilian SUS completed 31 years. Throughout its existence, it has already demonstrated capacity for the mission to reduce maternal mortality rates to the levels proposed at the Millennium Summit in 2000, albeit with a delay of a decade. Undoubtedly, the Ministry of Health needs to count on the participation of the entire organized society for this task. Initiatives such as the creation of the National Association for Safe and Respectful Childbirth, involving dozens of institutions such as Universities, Medical Societies, Hospital, Government Agencies, Non-Governmental Organizations, Unions, etc. is extremely important and can make a difference. However, there must be a centralized coordination of the actions to avoid the occurrence of the same errors observed during the Covid-19 pandemic, when the lack of this coordination led to a generalized diversification of conduct, where each municipality or each state made protocols their own way. Brazil, as an economic power, can offer comprehensive care compatible with the needs of Brazilian women and move away from the statistics that profile it alongside economically much poorer nations.
